# Characterization of Wildland Firefighters’ Exposure to Coarse, Fine, and Ultrafine Particles; Polycyclic Aromatic Hydrocarbons; and Metal(loid)s, and Estimation of Associated Health Risks

**DOI:** 10.3390/toxics12060422

**Published:** 2024-06-10

**Authors:** Joana Teixeira, Gabriel Sousa, Rui Azevedo, Agostinho Almeida, Cristina Delerue-Matos, Xianyu Wang, Alice Santos-Silva, Francisca Rodrigues, Marta Oliveira

**Affiliations:** 1 REQUIMTE/LAQV, Instituto Superior de Engenharia do Porto, Instituto Politécnico do Porto, R. Dr. António Bernardino de Almeida 431, 4249-015 Porto, Portugal; 2 REQUIMTE/UCIBIO, Unidade de Ciências Biomoleculares Aplicadas, Departamento de Ciências Biológicas, Faculdade de Farmácia, Universidade do Porto, R. Jorge de Viterbo Ferreira 228, 4050-313 Porto, Portugal; 3 REQUIMTE/LAQV, Departamento de Ciências Químicas, Faculdade de Farmácia, Universidade do Porto, R. Jorge Viterbo Ferreira, 228, 4050-313 Porto, Portugal; 4 QAEHS, Queensland Alliance for Environmental Health Sciences, The University of Queensland, 20 Cornwall Street, Woolloongabba, QLD 4102, Australia; 5 Laboratório Associado i4HB, Instituto para a Saúde e a Bioeconomia, Faculdade de Farmácia, Universidade do Porto, R. Jorge de Viterbo Ferreira 228, 4050-313 Porto, Portugal

**Keywords:** climate change, controlled fires, firefighting forces, inhalation exposure, fire emissions, health risk assessment

## Abstract

Firefighters’ occupational activity causes cancer, and the characterization of exposure during firefighting activities remains limited. This work characterizes, for the first time, firefighters’ exposure to (coarse/fine/ultrafine) particulate matter (PM) bound polycyclic aromatic hydrocarbons (PAHs) and metal(loid)s during prescribed fires, Fire 1 and Fire 2 (210 min). An impactor collected 14 PM fractions, the PM levels were determined by gravimetry, and the PM-bound PAHs and metal(loid)s were determined by chromatographic and spectroscopic methodologies, respectively. Firefighters were exposed to a total PM level of 1408.3 and 342.5 µg/m^3^ in Fire 1 and Fire 2, respectively; fine/ultrafine PM represented more than 90% of total PM. Total PM-bound PAHs (3260.2 ng/m^3^ in Fire 1; 412.1 ng/m^3^ in Fire 2) and metal(loid)s (660.8 ng/m^3^ versus 262.2 ng/m^3^), distributed between fine/ultrafine PM, contained 4.57–24.5% and 11.7–12.6% of (possible/probable) carcinogenic PAHs and metal(loid)s, respectively. Firefighters’ exposure to PM, PAHs, and metal(loid)s were below available occupational limits. The estimated carcinogenic risks associated with the inhalation of PM-bound PAHs (3.78 × 10^−9^ − 1.74 × 10^−6^) and metal(loid)s (1.50 × 10^−2^ − 2.37 × 10^−2^) were, respectively, below and 150–237 times higher than the acceptable risk level defined by the USEPA during 210 min of firefighting activity and assuming a 40-year career as a firefighter. Additional studies need to (1) explore exposure to (coarse/fine/ultrafine) PM, (2) assess health risks, (3) identify intervention needs, and (4) support regulatory agencies recommending mitigation procedures to reduce the impact of fire effluents on firefighters.

## 1. Introduction

Climate change promotes an increase in the number of occurrences and harshness of wildland fires, due to hotter and drier summer seasons across the world [1,2,3,4]. Unprecedented and severe wildfires, called large and mega-fires, are a reality in different areas of the world, including the Southern European countries (e.g., Greece, Spain, Italy, and Portugal), Australia, and some regions in the United States of America (e.g., California and Arizona) [5,6,7,8,9]. Wildfire mitigation is a world concern, and controlled forest fires and prescribed burns are tools frequently used for fire prevention and forest management. Controlled forest fires significantly reduce the forest fuel load and create a clean forest area that will protect local populations through the prevention of fire progression, thus avoiding the occurrence of uncontrolled large wildfires [10,11].

Wildfire emissions cause a health burden on exposed populations and firefighting forces through short- and long-term health impacts, including dehydration, headaches, extreme fatigue, dizziness, chronic inflammation, and the aggravation of a wide spectrum of respiratory (e.g., chronic obstructive pulmonary disease, hypoxia, and asthma) and cardiovascular (e.g., hypertension, heart disorders, myocardial infarction, and strokes) diseases and cancer (e.g., mesothelioma, melanoma and bladder cancer) [9,12,13,14,15,16,17,18]. Particulate matter (PM); carbon monoxide and dioxide; nitrogen oxides; metals; and several volatile organic compounds, including polycyclic aromatic hydrocarbons (PAHs), are some of the most common air pollutants found in fire emissions [19,20,21,22]. PM refers to airborne solid particles and liquid droplets of various sizes (coarse, PM > 2.5 µm of aerodynamic diameter; fine, 0.1 µm ≤ PM ≤ 2.5 µm; and ultrafine, PM ≤ 0.1 µm), shapes, and morphology [12,23,24,25,26]. PM can be originated from natural (e.g., forest fires, volcanic eruptions, and dust storms) or anthropogenic (e.g., vehicle exhausts, industrial emissions, heating and cooking systems, etc.) sources [12,23,24,25,26]. Ambient PM is carcinogenic to humans, and evidence exists for (probable/possible) carcinogens, including some metal(loid)s [e.g., arsenic (As), methylmercury, cadmium (Cd), nickel (Ni), few species of cobalt, trivalent antimony (Sb), and lead (Pb)], and PAHs [naphthalene, anthracene, benz(*a*)anthracene, benzo(*a*)pyrene (BaP), chrysene, benzofluoranthene isomers, dibenz(*a*,*h*)anthracene, indeno(1,2,3-c,d)pyrene, and dibenzo(*a*,*l*)pyrene] absorbed/adsorbed on its surface [27,28,29]. Inhalation of PM, mainly the fine and ultrafine fractions have the ability to generate reactive oxygen species that promote inflammation, oxidative stress, and cytotoxicity that may cause DNA mutations in the human respiratory system [13,30,31,32,33,34,35,36,37,38]. The ultrafine PM can reach the deeper parts of human lungs and even enter the bloodstream, being responsible for the development/aggravation of cardiorespiratory diseases, and lung cancer [12,13,14,15].

Occupational exposure as a firefighter was recently classified as carcinogenic to humans since it causes mesothelioma and bladder cancer [39]. Limited evidence suggests the development of colon, testicular, and prostate cancer, melanoma, and non-Hodgkin’s lymphoma in firefighters [39]. The available literature mostly characterizes PM_2.5_ levels released during wildfires and controlled forest fires, with data mostly emerging from the United States of America, Brazil, Australia, Africa, and some Asian countries [40,41,42,43,44,45,46,47,48,49,50]; scarce information is available for European countries [51,52]. Some authors already assessed the PM composition on organic [40,42,43,45,46,47,48,50,53] and inorganic [40,42,44,48,49,50,53] pollutants. Information related to firefighters’ exposure to ultrafine PM and its composition on organic and inorganic compounds during fire combat is scarce [50]. To reply to these scientific gaps, this work aims to characterize, for the first time, the exposure of Portuguese wildland firefighters to coarse, fine, and ultrafine PM during prescribed fires and its composition on 18 PAHs and 18 metal(loid)s, as well as estimate the associated health risks.

## 2. Materials and Methods

### 2.1. Prescribed Fires

Controlled forest fires, usually called prescribed fires, are commonly conducted during the winter season in areas that are previously identified by local authorities as having an increased risk for favoring the occurrence of large wildfires during the hot season. The prescribed fires are used to prevent wildfires through the reduction of forest fuel by creating areas with no vegetation that will represent a barrier to the propagation of fires, thus protecting the civil populations living in the surrounding areas.

**Figure 1:**
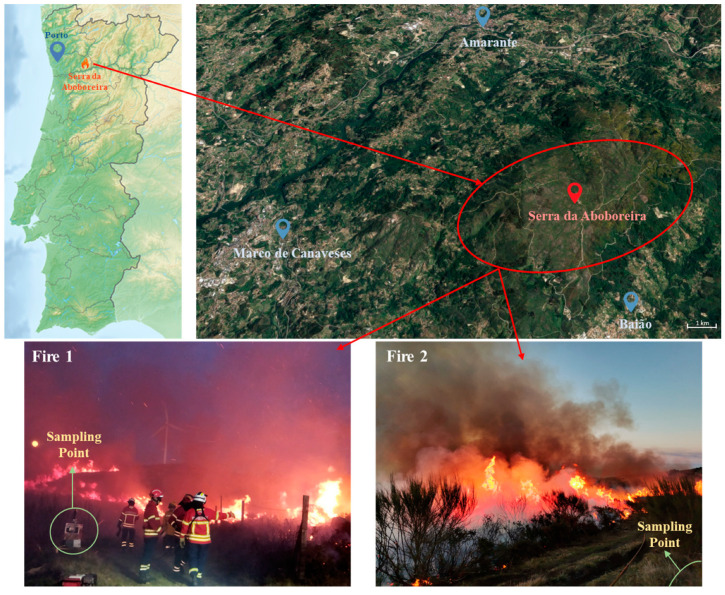
Geographical localization of Serra da Aboboreira (Porto, north of Portugal) where prescribed Fire 1 and Fire 2 occurred.

Serra da Aboboreira is a granite buttress located at the western end of the Marão/Alvão mountain massif, distributed across the municipalities of Marco de Canaveses, Baião, and Amarante, in the extreme northeast of the district of Porto, Portugal. This forest area is extensive, surrounded by small villages, and it was identified by local agents of civil protection and firefighters as an area with an increased risk for wildfires during the hot season. The selected forest area presented dry vegetation, mostly bushes (heather (*Calluna vulgaris*), scotch broom (*Cytisus scoparius*), and ulex (*Ulex europaeus*)) and small trees (eucalyptus (*Eucalyptus globulus*) and oak trees (*Quercus robur*)) with a propensity for a forest fire if favorable meteorological conditions occurred, endangering the nearby populations, fauna, and flora. As a preventive measure, two prescribed fires, Fire 1 and Fire 2, were planned by local authorities, and the dried forest fuel was ignited by the forest sappers, with a mixture of gasoline and diesel, during the cold season (January 2022) in Marco de Canaveses (Figure 1). Both controlled fires burned approximately 2000 m^2^. Two teams of 5 firefighters from the Marco de Canaveses and Porto fire stations, 5 forest sappers, and 2 local National Authority for Emergency and Civil Protection members were enrolled in Fire 1 and Fire 2 (210 min; 3.5 h). Overall, the intervention team members were all male (except for 1 female firefighter), 30–45 years old, with an estimated body mass index between 23.1 and 35.6 kg/m^2^ and with 7.3–26.1 years of activity as firefighters. All firefighters used the full personal protective equipment required for forest firefighting activities. The local temperature and relative humidity observed during both fire events were monitored in local meteorological stations and varied between 7 and 13 °C and between 32 and 45%, respectively. The wind speed ranged from 3.6 to 7.2 km/h, blowing in the north direction, and no precipitation was registered on the fire-event days.

### 2.2. Collection of PM

The breathable air of firefighters was collected with a cascade impactor (DLPI+, Dekati^®^, Kangasala, Finland) coupled with a vacuum pump (Leybold, Sogevac, Germany) at a safe distance within the firefighters’ working area and within 3 m of the fire’s front line. The sampling equipment was previously calibrated by fabricant and worked according to the supplier’s specifications at a constant pressure (40 mbar) and flow rate (9.96 L/min). The impactor was positioned at a height of 1.5 m above the ground to represent the breathing area of firefighters. The ambient temperature and humidity were monitored with a Hygrolog Serie HL20 (Rotronic, Switzerland) device.

In each sampling campaign, 14 fractions of PM were collected on aluminum filters (Ø25 mm; Dekati^®^, Kangasala, Finland) previously covered with grease (Apiezon^®^-L, Sigma-Aldrich, Steinheim, Germany) to prevent particle bounce. The 14 collected stages included 4 coarse (particles with a cut-off diameter of 9.88, 5.36, 3.65, and 2.47 µm), 6 fine (particles with 1.63, 0.947, 0.603, 0.382, 0.256, and 0.156 µm), and 4 ultrafine (particles with 0.095, 0.054, 0.031, and 0.0149 µm) PM fractions. Field-site travel blank filters were collected on each sampling day and treated as a sample. The mass of PM from each fraction was determined via a gravimetric analysis, i.e., by weighting filters before and after sampling, in an analytical balance (MS205DU, Mettler Toledo, Hong Kong, China), at a controlled temperature and relative humidity [54,55]. The filters were coded and stored in individual cartridges at −20 °C until further analysis.

### 2.3. Quantification of PAHs

Half of each PM filter was extracted with 10 mL of acetonitrile in an ultrasonic bath (Sonorex Digital 10P, Bandelin, Germany) for 20 min, at room temperature. The extracts were evaporated in a rotary evaporator (Rotavapor R-200, Büchi Labortechnik AG, Switzerland) at room temperature till dryness. Each extract was reconstituted in 500 µL of acetonitrile and filtered with a polytetrafluoroethylene filter (0.22 µm) before the chromatographic analysis.

The prepared organic extracts were analyzed in a Shimadzu LC apparatus (Shimadzu Corporation, Kyoto, Japan) equipped with a degasser (DGU-20AS), a photodiode array (SPD-M20A), and a fluorescence (RF-10AXL) detector in a C_18_ column (EC 150/4 Nucleosil 100–5 C18 PAH, 150 × 4.0 mm; 5 μm particle size, from Macherey–Nagel, Germany) at 25 °C [56,57]. Calibration curves were prepared using 6 calibration points of a mixture containing 18 compounds: naphthalene, acenaphthylene, acenaphthene, fluorene, phenanthrene, anthracene, fluoranthene, pyrene, benz(a)anthracene, chrysene, benzo(b + j)fluoranthene, benzo(k)fluoranthene, BaP, dibenzo(a,l)pyrene, dibenz(a,h)anthracene, benzo(g,h,i)perylene, and indeno(1,2,3-c,d)pyrene). The limits of detection (LODs) were determined as the minimum detectable amount of a compound with a signal-to-noise ratio of 3:1 [58]. The LOD values ranged between 0.0652 µg/L for benz(a)anthracene and 3.72 µg/L for acenaphthylene. The methodology applied achieved recoveries between 13.5% for acenaphthylene and 99.2% for dibenz(a,h)anthracene. The standards, blanks, and samples were daily analyzed in triplicate.

### 2.4. Quantification of Metal(loid)s

The other half of the PM filter was submerged in 10 mL of acetone (Sigma Aldrich, Steinheim, Germany) and sonicated for 20 min at room temperature. The extract containing the collected particles was evaporated to dryness. An aliquot of 3 mL of nitric acid (≥65%, Sigma Aldrich, Steinheim, Germany) and 1.5 mL of hydrogen peroxide (30%, Merck Millipore, Burlington, MA, USA) were added, and the mixture was digested in DigiPREp Jr (SCP Science, Baie D’Urfé, QC, Canada) equipment during 30 min, at 95 °C. The digested extract was diluted with ultrapure water (>18.2 MΩ·cm at 25 °C, Arium^®^ pro, Sartorius, Göttingen, Germany) to 20 mL. An aliquot of 5 mL of a solution containing 0.5 mL of the diluted extract and 4.5 mL of diluent solution (2% (*v*/*v*) nitric acid (67–69% *w*/*w* TraceMetal^®^ Grade, Fisher Scientific, Hanover Park, IL, USA), 500 µg/L gold (TraceSELECT^®^, Fluka, Seelze, Germany),1.5% (*v*/*v*) ethanol absolute anhydrous and 10 µg/L internal standard (Internal Standard Mix 1—SCP-IS7, PlasmaCAL^®^, SCP Science)) was analyzed by inductively coupled plasma–mass spectrometry, using an iCAP™ Q (Thermo Fisher Scientific, Bermen, Germany) instrument and according the methods provided by the National Institute for Occupational and Health (NIOSH) and the United States Environmental Protection Agency (USEPA) [59,60]. The mineral contents of 18 metal(loid)s, lithium (Li), beryllium (Be), chromium (Cr), cobalt (Co), Ni, copper (Cu), zinc (Zn), As, selenium (Se), strontium (Sr), molybdenum (Mo), Cd, Sb, cesium (Cs), barium (Ba), mercury (Hg), thallium (Tl), and Pb, were analyzed. The methodology presented recoveries ranging from 95.4% for Se to 118.1% for Zn. The calculated LOD values ranged between 3.4 ng/L (Be) and 2.08 µg/L (Cu). All samples and standards were analyzed in triplicate.

### 2.5. Health-Risk Analysis

A human health-risk assessment estimates the nature and probability of adverse health effects in humans who were exposed to chemicals in contaminated media. USEPA pioneered a health-risk methodology based on scientific data on human exposure-to-dose relationships and the consequent health response in humans [61,62,63]. This methodology has been widely applied to estimate the health risks related to human environmental/occupational exposure to hazardous pollutants, including PAHs and heavy metals) [31,64,65,66,67,68,69,70]. Following the previous works of these authors [31,65], the USEPA methodology was used to perform a firefighters’ health-risk analysis associated with the respirable PM-bound PAHs and metal(loid)s during the two monitored controlled fires.

The health-risk assessment was estimated through the determination of carcinogenic and non-carcinogenic risks by using the concentrations of PM-bound PAHs and metal(loid)s quantified in the air of firefighters during the fire events. Inhalation’s total carcinogenic risk (TR, dimensionless) and the target hazard quotient (THQ, dimensionless) were estimated according to the methodology provided by USEPA [61], which is briefly described.

The TR estimates the accumulative probability of an individual developing cancer during his/her lifetime due to exposure to a specific carcinogenic compound. TRs were determined according to Equation (1):

(1) $$TR=\frac{{EF}_{R}\times ED\times ET\times IUR\times C}{AT}$$

The following assumptions were made: EF_R_ is the exposure frequency (250 days of exposure per year, i.e., participation in 1 fire event (controlled/real) per working day as the annually mean exposure baseline); ED is the exposure duration, and it was considered the reported average length of the participants’ career (15.4 years) since it represents the occupational activity already made by each participant; ET is the exposure time monitored in this work, corresponding to the duration of the controlled fire event (3.5 h/day); IUR is the chronic inhalation unit risk [(μg/m^3^)^−1^] retrieved from the USEPA risk-based concentration table [71]; C is the concentration of PAH or metal considered (μg/m^3^); and AT is the number of days over which the exposure is averaged during the expected firefighter career (14,600 days, i.e., 40 years of career × 365 days/year [71]). TR values were calculated for PAHs and metal(loid)s with available IUR values: naphthalene (3.4 × 10^−5^ μg/m^3^); benz(a)anthracene and indeno(1,2,3–c,d)pyrene (6.0 × 10^−5^ μg/m^3^); chrysene (6.0 × 10^−7^ μg/m^3^); benzo(k)fluoranthene (6.0 × 10^−6^ μg/m^3^); BaP and dibenzo(a,h)anthracene (6.0 × 10^−4^ μg/m^3^); benzo(b)fluoranthene (1.1 × 10^−4^ μg/m^3^); As (4.3 × 10^−3^ μg/m^3^); Cr (8.4 × 10^−2^ μg/m^3^); Co (9.0 × 10^−3^ μg/m^3^); and Ni (2.4 × 10^−4^ μg/m^3^). Supplementary Table S1 presents an example for the calculation of TR. The TR risk is acceptable if calculated values range between 10^−4^ (risk of developing cancer over a human lifetime is 1 in 10,000) and 10^−6^ (risk of developing cancer over a human lifetime is 1 in 1,000,000) [63].

THQ estimates the non-carcinogenic risk of a compound, and it was determined with Equation (2).

(2) $$THQ=\frac{{EF}_{R}\times ED\times ET\times C\times I_{R}}{{Rf}_{D}\times AT\times B_{W}}$$

The variables EF_R_, ED, ET, AT, and C were already defined for TR. I_R_ is the age-specific weighted average breathing rate considering the age range and physical intensity level of the activity performed by the firefighters enrolled in the study (1.25 × 10^−2^ m^3^/min; light intensity). B_W_ is the age-specific body weight of participants (~83.7 kg). Rf_D_ is the reference dose (mg/kg day) for each PAH and metal analyzed: acenaphthene (6.0 × 10^−2^ mg/kg day); anthracene (3.0 × 10^−1^ mg/kg day); fluoranthene and fluorene (4.0 × 10^−2^ mg/kg day); naphthalene (2.0 × 10^−2^ mg/kg day); pyrene (3.0 × 10^−2^ mg/kg day); Al (1.0 mg/kg day); As, Co and Sb (3.0 × 10^−4^ mg/kg day); Ba (2.0 × 10^−1^ mg/kg day); Cr (3.0 × 10^−3^ mg/kg day); Cu (4.0 × 10^−2^ mg/kg day); Fe (7.0 × 10^−1^ mg/kg day); Li (2.0 × 10^−3^ mg/kg day); Mn (2.4 × 10^−2^ mg/kg day); Mo (5.0 × 10^−3^ mg/kg day); Sr (6.0 × 10^−1^ mg/kg day); Zn (3.0 × 10^−1^ mg/kg day); and Ni (1.1 × 10^−2^ mg/kg day) [71]. THQ values higher than unity suggest the existence of non-carcinogenic effects, while values below the unity represent negligible non-carcinogenic risks.

### 2.6. Data Treatment

The levels of PM were corrected for ambient temperature (21 °C), humidity, and atmospheric pressure, as recommended by the supplier of impactor. The concentrations of total PM were calculated based on the total mass of particles collected in the 14 fractions and the normalized sampled air volume. The results are present as median and range since the values do not follow a normal distribution. Whenever a compound was not detected in the PM fraction, the concentration was replaced by LOD/√2 [72]. The treatment of generated data and statistical analysis was accomplished using Microsoft Excel (Microsoft Corporation, Washington, DC, USA) and SPSS (IBM SPSS Statistics 20). Results are presented as median and range since the data do not follow a normal distribution, demonstrated by the Shapiro–Wilk test. The Mann–Whitney U test was used to compare the results with a statistical significance defined as *p* ≤ 0.05.

## 3. Results

### 3.1. Levels of PM

The levels of total cumulative PM were four times higher in Fire 1 than in Fire 2 (1408.3 versus 342.5 µg/m^3^), evidence that can be attributed to the fuel load available in the selected areas and changes in the wind direction and intensity that conditioned the progression and direction of fire plumes toward firefighters. The fine PM was the predominant fraction (1133.0 µg/m^3^ in Fire 1 and 225.0 µg/m^3^ in Fire 2; 80.45% and 65.71% of total cumulative PM, respectively), followed by ultrafine PM (165.2 µg/m^3^ in Fire 1 and 107.6 µg/m^3^ for Fire 2; 11.73% and 31.43%, respectively). Altogether, fine and ultrafine PM represented more than 90% of total PM (Supplementary Figure S1). The coarse PM, 110.1 µg/m^3^ in Fire 1 and 9.78 µg/m^3^ in Fire 2, accounted for 7.82% and 2.86% of total PM (Figure S1). The median levels of non-cumulative PM_10_ and PM_2.5_ monitored during both prescribed fires were 33.4 and 243.0 µg/m^3^ (Fire 1), and 3 and 115.3 µg/m^3^ (Fire 2), respectively.

### 3.2. Concentrations of PM-Bound PAHs

During prescribed Fires 1 and 2, levels of total PM-bound PAHs of 3260.2 ng/m^3^ and 412.1 ng/m^3^ were found in the air of Portuguese firefighters, respectively. The concentrations of total and individual PAHs bounded to (coarse, fine, and ultrafine) PM are presented in Table 1. The PAH levels were superior in fine PM (2719.0 ng/m^3^ for Fire 1 and 206.1 ng/m^3^ for Fire 2; *p* < 0.001), followed by ultrafine (436.6 versus 106.8 ng/m^3^; *p* = 0.493) and coarse PM (104.6 versus 99.2 ng/m^3^; *p* < 0.001). A similar tendency was observed when the PM-bound PAH levels were expressed by mass of collected particles; however, ultrafine PM presented increased levels of total PAHs compared to fine and coarse PM (Supplementary Table S3).

**Figure 2:**
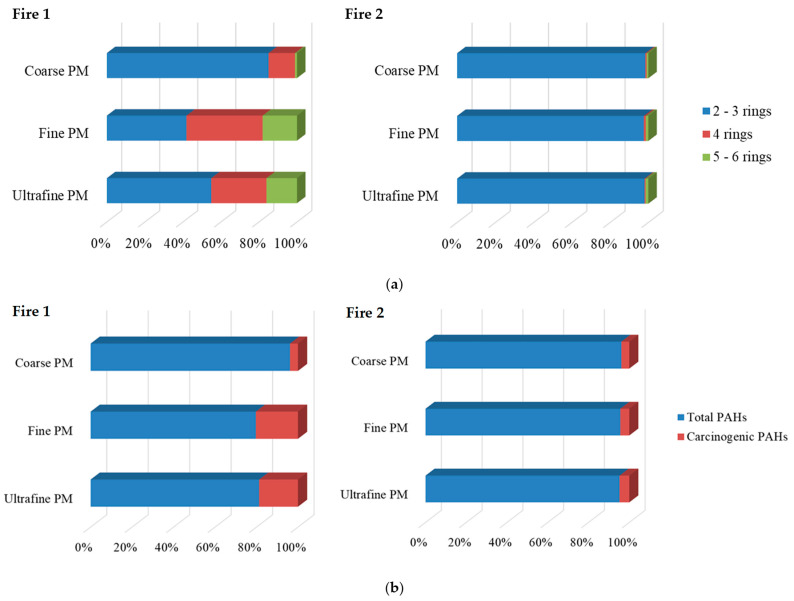
Distribution of PAHs according to (**a**) the number of aromatic rings during prescribed fires and (**b**) total PAHs and total carcinogenic PAHs in Fires 1 and 2.

Out of the 18 PAHs under study, only acenaphthene and dibenzo(a,l)pyrene were not detected in Fire 1, while acenaphthene, anthracene, benz(a)anthracene, benzofluoranthene isomers, BaP, dibenzo(a,h)anthracene, and benzo(g,h,i)perylene were not detected in Fire 2 (Table 1). Overall, the low-molecular-weight PAHs (two or three rings) were the prevalent compounds in the coarse (85.1% and 98.4% of total PAHs for Fire 1 and Fire 2), fine (41.9% and 97.6% of total PAHs), and ultrafine (54.9% and 98.0% of total PAHs) PM released during the prescribed fires (Figure 2a). Compounds with four (13.9%, 40.1%, and 29.1% in coarse, fine, and ultrafine PM of Fire 1; 0.69%, 1.07%, and 0.50% in coarse, fine, and ultrafine PM of Fire 2) and five or six aromatic rings (1.06%, 18.1%, and 16.1% in coarse, fine, and ultrafine PM of Fire 1; 0.88%, 1.29%, and 1.46% in coarse, fine, and ultrafine PM of Fire 2) were the less abundant (Figure 2a). 

**Figure 3:**
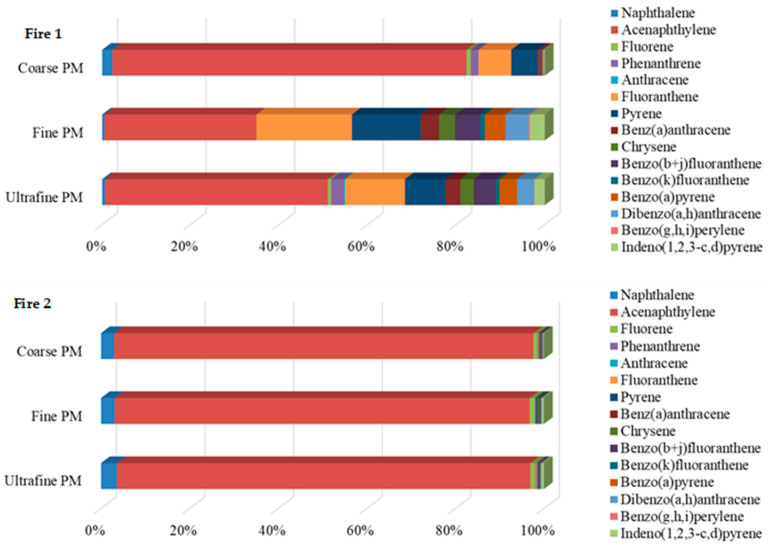
Distribution of PAHs in the coarse, fine, and ultrafine PM during prescribed Fires 1 and 2.

Acenaphthylene (832.9 ng/m^3^ in fine, 219.7 ng/m^3^ in ultrafine, and 83.7 ng/m^3^ in coarse PM), fluoranthene (523.0 ng/m^3^ in fine, 58.9 ng/m^3^ in ultrafine, and 7.69 ng/m^3^ in coarse PM), and pyrene (379.0 ng/m^3^ in fine, 40.5 ng/m^3^ in ultrafine, and 6.20 ng/m^3^ in coarse PM) were the predominant compounds in the PM collected in the air of firefighters during Fire 1 (Table 1). Altogether, these three compounds accounted for 93.3%, 73.1%, and 63.7% of total PAHs in the coarse, ultrafine, and fine PM, respectively (Figure 3). Regarding Fire 2, acenaphthylene (191.9 ng/m^3^ in fine PM, 99.4 ng/m^3^ in ultrafine PM, and 93.7 ng/m^3^ in coarse PM), naphthalene (6.41 ng/m^3^ in fine, 3.83 ng/m^3^ in ultrafine, and 2.96 ng/m^3^ in coarse PM), and fluorene (2.40 ng/m^3^ in fine, 1.20 ng/m^3^ in ultrafine, and 0.795 ng/m^3^ in coarse PM) were the most abundant compounds and represented 98.3%, 97.8%, and 97.4% of the total PAHs in the coarse, ultrafine, and fine PM, respectively (Table 1; Figure 3). Anthracene (0.0667–37.3 ng/m^3^), benz(a)anthracene (0.400–99.0 ng/m^3^), benzofluoranthene isomers (0.0564–135.0 ng/m^3^), BaP (0.284–114.4 ng/m^3^), and benzo(g,h,i)perylene (0.157–6.93 ng/m^3^) were detected only in samples from Fire 1, while dibenzo(a,l)pyrene (0.78–0.498 ng/m^3^) was detected only in fine PM from Fire 2 (Table 1).

The (possible/probable) carcinogenic PAHs were found in the PM released from both fires, with total concentrations of 797.7 ng/m^3^ (i.e., 24.5% of total PAHs) in Fire 1 and 18.8 ng/m^3^ (i.e., 4.57% of total PAHs) in Fire 2. Concerning the distribution among the PM phases, concentrations of carcinogenic PAHs were increased in fine (692.9 and 9.48 ng/m^3^ in Fires 1 and 2, respectively) compared to ultrafine (100.6 ng/m^3^ versus 5.35 ng/m^3^) and coarse (4.18 ng/m^3^ versus 3.99 ng/m^3^) PM (Figure 2b). BaP was only detected in the air of Fire 1, with increased levels in fine (114.4 ng/m^3^) compared to ultrafine (17.3 ng/m^3^) and coarse (0.28 ng/m^3^) PM (Table 1).

### 3.3. Concentrations of PM-Bound Metal(loid)s

The levels of total PM-bound metal(loid)s in the air of firefighters were 660.8 ng/m^3^ (643.5–686.2 ng/m^3^) in Fire 1 and 262.2 ng/m^3^ (251.1–277.0 ng/m^3^) in Fire 2 (Table 2). The metal content was predominantly increased in fine PM (420.0 ng/m^3^ in Fire 1 and 123.1 ng/m^3^ in Fire 2; *p* = 0.115) than in the ultrafine PM (119.1 versus 89.56 ng/m^3^; *p* > 0.05) (Table 2). The lowest values of PM-bound metal(loid)s were found in the coarse fraction of Fire 2 (49.61 ng/m^3^) (Table 2). Overall, the fine PM (63.6% in Fire 1 and 46.9% in Fire 2) contributed more to total metal content than ultrafine (18.0% versus 34.2%) and coarse (18.4% versus 18.9%) PM (Table 2). However, when the levels of PM-bound metal(loid)s were expressed per mass of collected particles, the highest concentrations were observed in ultrafine fraction, as also observed for PM-bound PAHs (Supplementary Tables S3 and S5).

**Figure 4:**
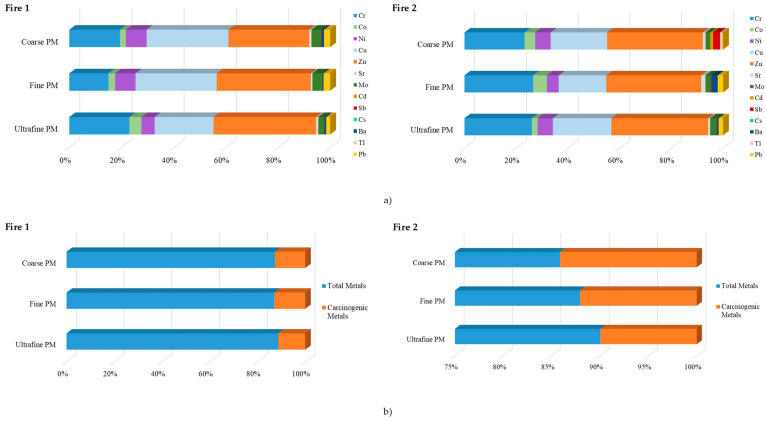
Distribution of (**a**) metals in the coarse, fine, and ultrafine PM and (**b**) total and carcinogenic metals during prescribed fires.

Among the different metal(loid)s under analysis, Li, Be, As, Se, and Hg were never detected in the PM of Fires 1 and 2. Zn, Cu, and Cr were the most abundant elements in all the PM fractions from both fire events (Zn: 37.6, 151.5, and 46.8 ng/m^3^ versus 18.4, 45.0, and 33.4 ng/m^3^ in the coarse, fine, and ultrafine PM of Fires 1 and 2, respectively; Cu: 38.1, 130.3, and 26.9 ng/m^3^ versus 10.8, 22.5, and 20.2 ng/m^3^; Cr: 23.5, 62.8, and 27.4 ng/m^3^ versus 11.5, 32.5, and 23.2 ng/m^3^) (Table 2). These three metals represent 81.5–82.0%, 81.3–82.1%, and 84.9–85.8% of total coarse, fine, and ultrafine PM-bound metals in Fires 1 and 2 (Figure 4a). Cd, Cs, and Tl were the least abundant PM-bound metals in both fires (Table 2).

The concentrations of total PM-bound (possible/probable) carcinogenic metal(loid)s (Co, Ni, Cd, Sb, and Pb) were increased in Fire 1 compared to Fire 2 (83.5 versus 30.8 ng/m^3^) and represented 12.6% and 11.7% of total PM, respectively (Table 2). The levels of carcinogenic metal(loid)s were increased in fine PM (54.58 ng/m^3^ in Fire 1 and 14.85 ng/m^3^ in Fire 2) than in ultrafine (13.49 ng/m^3^ versus 8.924 ng/m^3^) and coarse (15.45 ng/m^3^ versus 7.017 ng/m^3^) PM (Table 2), accounting for 11.3–13.0% and 9.96–14.1% of total PM (Figure 4b).

### 3.4. Firefighter’s Health Risk Evaluation

**Figure 5:**
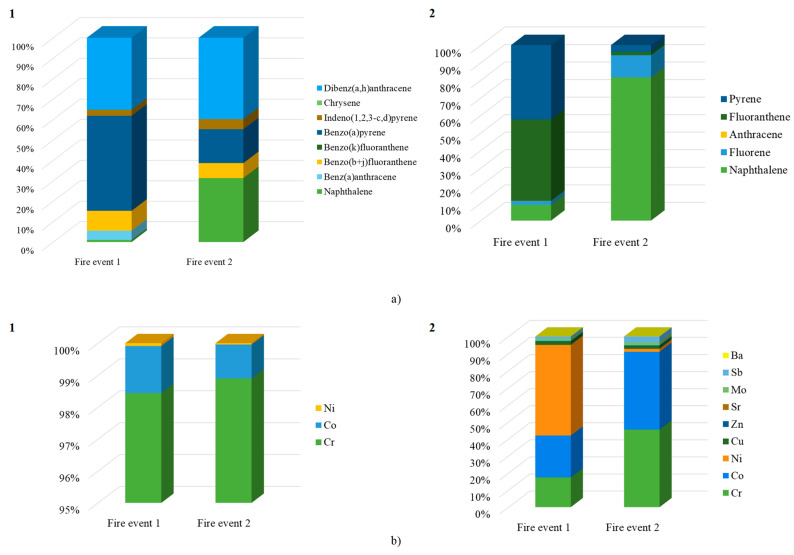
Contribution of (**a**) PM-bound PAHs (%) and (**b**) PM-bound metals (%) to (1) total TR and (2) total THQ values due to inhalation of PM during prescribed Fire 1 and 2.

The estimated carcinogenic risks associated with the inhalation of PM-bound PAHs during the monitored prescribed fires ranged between 9.52 × 10^−9^ and 4.39 × 10^−6^. Concerning individual TR values, BaP (46.5% in Fire 1 and 16.5% in Fire 2), dibenz(a,h)anthracene (35.2% in Fire 1 and 39.8% in Fire 2), benzo(b + j)fluoranthene (9.70% versus 7.32%, respectively), and naphthalene (1.02% versus 31.3% in Fire 1 and 2, respectively), were the major contributors to total TR during controlled fire events (Figure 5a1). Firefighters’ total TR values are presented in Supplementary Table S7. As expected, increased values of total TR were obtained for fine and ultrafine PM. Overall, total TRs values were increased for Fire 1 compared to Fire 2 (1.08 × 10^−8^ versus 7.68 × 10^−9^, 1.47 × 10^−6^ versus 8.10 × 10^−9^, and 3.04 × 10^−7^ versus 8.16 × 10^−9^ for coarse, fine, and ultrafine PM, respectively).

Relative to airborne PM-bound metal(loid)s released during prescribed fires, estimated carcinogenic risks varied between 1.50 × 10^−2^ and 2.37 × 10^−2^. Cr contributed 98.4% and 98.9% of total TR in Fires 1 and 2, followed by Co (1.47% versus 1.06%) and Ni (0.085% versus 0.039%) (Figure 5b1). Regarding the total TR due to exposure to metal(loid)s, a similar tendency was observed, with increased carcinogenic risk being observed for firefighters enrolled in Fire 1 than in Fire 2 (2.71 × 10^−2^ versus 1.09 × 10^−2^ for coarse, 2.58 × 10^−2^ versus 1.41 × 10^−2^ for fine, and 2.33 × 10^−2^ versus 1.89 × 10^−2^ for ultrafine PM; Supplementary Table S7).

The determined non-carcinogenic risks due to the inhalation of PM-bound PAHs and metal(loid)s ranged between 4.77 × 10^−7^ and 9.94 × 10^−5^ and from 1.17 × 10^−2^ to 2.93 × 10^−1^, respectively. Concerning individual PAHs, naphthalene (9.01–81.5%), fluoranthene (1.92–46.4%), and pyrene (3.83–42.4%) were the major contributors to total THQ in both prescribed fires (Figure 5a2). Regarding PM-bound metals, Cr (17.5–45.5%), Co (24.5–45.5%), and Ni (1.70–53.0%) were the main contributors to total THQ (Figure 5b2) in firefighters.

## 4. Discussion

### 4.1. Levels of PM

During a prescribed fire, firefighters control the fire line and position themselves against the wind direction to avoid exposure to fire emissions; however, changes in local meteorological conditions (temperature, relative humidity, and/or wind direction/intensity) can potentiate hot spots where moderate exposure to fire emissions might occur. The four-times-increased PM concentrations in Fire 1 comparatively to Fire 2 (1408.3 versus 342.5 µg/m^3^, respectively) were primarily attributed to the denser and taller vegetation, and secondly to the lower steepness observed in the pre-selected area. Despite being performed in the same mountain with the same predominant species, some changes were observed in the topology and composition of fuel in the two pre-selected areas. Additionally, some changes were observed in the wind direction during Fire 1, which favored the progression of fire emissions toward the sampling point. The occupational exposure limit defined by the Occupational Safety and Health Organization (OSHA) for respirable dust, 5.0 mg/m^3^, was not overcome during both fire events. The levels of total PM observed in these fires were aligned with the values (50.12 and 27,211 µg/m^3^) reported by other authors in a prescribed and a live fire, respectively [73]. The PM_2.5_ levels are aligned with the values (overall range: 8.8–264 µg/m^3^) described by different authors [74,75,76,77,78] but lower than the range of concentrations (354–8357 µg/m^3^) reported by other authors in wildfires and prescribed burns [79,80,81,82,83] (Supplementary Table S2). The variability observed among the different studies can be attributed to the wide variety of fuels burned and the atmospheric conditions in the burnt area, as well as to the use of distinct sampling methods used in each study (Supplementary Table S2). Ambient relative humidity also affects the moisture content of the vegetation and the forest fuel load, consequently impacting PM emissions [82]. A higher wind velocity contributes to the widespread of smaller/lighter particles over longer distances, while the combustion of drier and smaller vegetation (e.g., reed, gorse and other shrubs) results in the emission of smaller particles [84], and the combustion of vegetation with superior dimensions (e.g., trees of pines, eucalyptus, and oak) emits an inferior concentration of PM, with a predominance of particles with superior size [85,86].

Over the last few years, some concern has emerged about the contribution of prescribed fires to the deterioration of the ambient air quality, thus contributing to the health burden of local populations [17,87,88,89]. The World Health Organization recently updated the recommended PM ambient air-quality guidelines (45 and 15 µg/m^3^ for short-term exposure (daily basis) and 15 and 5 µg/m^3^ for long-term exposure (annual basis) to PM_10_ and PM_2.5_, respectively) based on the literature reviewed [23]. The levels of PM_10_ and PM_2.5_ monitored during both prescribed fires are within the recommended guideline for short-term exposure to PM_10_ but exceeded 16 times the ambient PM_2.5_ recommendation. These findings highlight the potential contribution of prescribed fires to ambient air pollution.

### 4.2. Concentrations of PM-Bound PAHs

Firefighters’ exposure to airborne PAHs while working at prescribed fires did not exceed the occupational limits defined by OSHA (200 µg/m^3^) and NIOSH (100 µg/m^3^) [90,91]. To the best knowledge of these authors, this is the first study to simultaneously assess the levels of coarse, fine, and ultrafine PM-bound PAHs during prescribed fires. The available literature mostly characterized the PAH levels on total PM during prescribed fires and wildfires and reported predominantly higher concentrations (overall range: 73–9103 ng/m^3^) [75,92]. Some authors also described the prevalence of low-molecular-weight PAHs in the fire emissions of prescribed burns and wildfires [40,76,88,92,93,94,95]. The concentrations found in this study were predominantly higher than the levels of fine PM-bound benzofluoranthene isomers (162.7 ng/m^3^ versus 73.0 ng/m^3^) and phenanthrene (0.50–236.4 ng/m^3^ versus 132.0 ng/m^3^) reported by [75]; however, they were inferior to the values of fine PM-bound fluorene (2.40–17.5 ng/m^3^ versus 830.0 ng/m^3^), phenanthrene (0.50–236.4 ng/m^3^ versus 640.0 ng/m^3^), and naphthalene (6.41–13.9 ng/m^3^ versus 6170 ng/m^3^) presented by [41]. The levels of cumulative PM_2.5_-bounded PAHs, i.e., sum of ultrafine and fine PM (3155.6 ng/m^3^ for Fire 1 and 312.9 ng/m^3^ for Fire 2) were within the range of values (overall range: 40–7640 ng/m^3^) presented by other studies characterizing prescribed and live fires (Supplementary Table S4). Overall, the concentrations of PM-bound BaP (131.98 ng/m^3^) accounted for 4.05% of total PAHs released in Fire 1 and were in close range with the values (3.00–185 ng/m^3^) reported by Robinson et al. (2008) in prescribed fires but slightly higher than the levels (overall range: 3.00–120 ng/m^3^) described by other authors in prescribed burns and wildfires [41,76,92,96]. The concentrations of total PM-bound BaP determined in the PM collected during prescribed fires largely exceeded the European and national ambient air-quality guideline of 1 ng/m^3^ [97,98]. These findings demonstrate the negative impact that prescribed fires and wildfires can have on local populations, as previously demonstrated by some authors [25,40,41,48,50,99,100]. Moreover, several studies in the literature demonstrate the increased levels of organic pollutants and/or their main metabolites (e.g., xylene, toluene, styrene, acrolein, PAHs, per- and polyfluoroalkyl substances, etc.) in the urine and blood of firefighters after their active participation in firefighting activities [13,101,102,103,104,105,106].

### 4.3. Concentrations of PM-Bound Metal(loid)s

The presence of different metals in the fumes of biomass combustions, including forest fires, was previously reported by other authors [40,42,48,107,108,109,110,111]. So far, limited information is available on the metal content of PM_2.5_ released during prescribed fires (Supplementary Table S6); no data are available for coarse and ultrafine PM. The concentrations of total PM_2.5_-bound metals, i.e., the sum of fine and ultrafine fractions of collected PM (539.1 ng/m^3^ in Fire 1 and 212.7 ng/m^3^ in Fire 2), determined in the air of firefighters during prescribed fires are below the range of values (2.311–54.10 µg/m^3^) reported in the literature (Supplementary Table S6). The concentrations of total PM-bound Ni (48.36 versus 13.83 ng/m^3^), Co (19.20 ng/m^3^ in Fire 1 and 10.57 ng/m^3^ in Fire 2), Pb (15.06 versus 4.194 ng/m^3^), Sb (0.7660 versus 1.621 ng/m^3^), and Cd (0.1927 versus 0.5871 ng/m^3^) were below the permissible airborne occupational exposure limits defined by OSHA (1.0 × 10^6^ ng/m^3^ for Ni, 0.1 × 10^6^ ng/m^3^ for Co, 50 × 10^3^ ng/m^3^ for Pb, 0.5 × 10^6^ ng/m^3^ for Sb, and 5 × 10^3^ ng/m^3^ for Cd over an 8 h work shift) [112,113,114,115,116,117]. The available literature reports predominantly increased levels of Co (4.71–18.1 ng/m^3^), Ni (102–277 ng/m^3^), Sb (537 ng/m^3^), Cd (4.95–16.1 ng/m^3^), and Pb (61.0–105 ng/m^3^) in the PM_2.5_ released from prescribed fires and wildfires [40,42,48]. The presence and variability of PM metals in fire emissions are directly related to burnt fuels, the composition of raw materials, the temperature reached in the fire, and the duration of the combustion process [118,119]. It is known that emissions from the burning of electric/electronic devices and materials containing plastics and/or paints (e.g., high-tension cables, agriculture vehicles and equipment, batteries, etc.), are expected to present increased levels of some metals (e.g., Pb, Cu, Li, or Cd) that can be found in the air of areas where wildland–urban interface fires, including prescribed fires, occurred [107,120,121,122].

### 4.4. Firefighter’s Health Risk Evaluation

Besides the use of USEPA methodology on ecological risk assessment [123,124], this methodology has been extensively applied in the determination of personal risks due to human exposure to hazardous compounds [31,64,65,66,67]. The estimated carcinogenic risks were predominantly below the USEPA’s acceptable risk level (10^−4^ and 10^−6^), while the carcinogenic risks related to airborne PM-bound metal(loid)s were 150–237 times higher than the USEPA’s maximum acceptable risk level (10^−4^). The non-carcinogenic risks due to the inhalation of PM-bound PAHs and metal(loid)s were below the unity defined by USEPA, and thus non-carcinogenic risks due to a firefighter’s participation in prescribed fires can be considered negligible. These findings highlight the existence of some carcinogenic risks to firefighters during their participation in prescribed fires. However, the assumptions assumed for the health-risk analysis reflect the working activity of Portuguese firefighters, which may not represent the reality of other firefighting forces. So far, limited studies focusing on firefighters’ health-risk analysis are available in the literature. Rakowska et al. [125] demonstrated the existence of carcinogenic and non-carcinogenic risks associated with Polish firefighters’ exposure to fire emissions in the garage of the fire station after the day of a firefighting operation (1.01 × 10^−4^–3.45 × 10^−4^ and 0.12–0.57, respectively); carcinogenic-risk values were up to 100 times higher than the levels estimated in this study. Similar results were reported by Valdenaire and co-authors [126] for Parisian sapper firefighters, with an estimated carcinogenic risk level varying between 2.25 × 10^−5^ and 1.16 × 10^−4^. More recently, Teixeira et al. [65] characterized Portuguese firefighters’ exposure to PAHs during firefighting activities and performed a comprehensive health-risk assessment that demonstrated the existence of significantly higher carcinogenic risks during firefighting comparatively with regular workdays at fire station (4.25 × 10^−5^–1.35 × 10^−4^ versus 7.56 × 10^−6^). Regarding firefighters’ exposure to PAHs at fire stations and associated carcinogenic risks, Oliveira et al. [31] characterized the personal exposure to PM_2.5_-bound PAHs inside five fire stations during regular working activities, excluding fire events. The authors reported carcinogenic risk levels ranging from 1.94 × 10^−8^ to 1.22 × 10^−7^, negligible values that are aligned with the findings of this study (1.47 × 10^−6^ and 8.10 × 10^−9^). Rogula-Kozlowska and co-authors [127] concluded that carcinogenic risks estimated for office workers and firefighters working inside two Polish fire stations were above the USEPA’s recommended level (2.16 × 10^−8^–2.06 × 10^−6^ and 4.30 × 10^−6^–5.81 × 10^−6^, respectively) and higher than the risks estimated during the monitored controlled forest fires.

To the best of the knowledge of these authors, studies performed with firefighters that included exposure to metal(loid)s and estimated the associated health risks are inexistent. Dehghani et al. [128] evaluated the health risks associated with the occupational exposure to heavy metals in a casting unit of a steel plant and reported carcinogenic risks for lead and non-carcinogenic risks for lead and manganese higher than the USEPA’s acceptable levels (10^−4^–10^−6^). Wongsasuluk and co-authors [129] carried out a health risk assessment related to the exposure to arsenic and some heavy metals in gold mines of Myanmar. The carcinogenic risks reported by those authors for Pb (6.72 × 10^−4^–1.23 × 10^−3^) were up to 12 timed higher than USEPA maximum risk level (10^−4^); the estimated non-carcinogenic risks were negligible. Overall, these authors demonstrate the carcinogenic risks associated with occupational exposure to metal(loid)s, being also aligned with the increased carcinogenic risks found in firefighters enrolled in controlled fires. However, additional studies, including health-risk analyses, are urgently needed to corroborate the findings of this study.

It is worth mentioning that the protection given by firefighters’ personal protective equipment was not evaluated in this study. Also, fire emissions release several other fire effluents besides PM, PAHs, and metals [13,19,20,22,102,106]; therefore, it is predictable that non-carcinogenic and carcinogenic risks for firefighters due to controlled and real firefighting activities during a regular working year are higher than the values reported in this study. The estimated health risks contribute to a more comprehensive assessment of firefighters’ health risks and need to be supported by complementary studies (e.g., biomonitoring analysis to determine the levels of biomarkers of exposure and/or effect, and in vitro assays to assess cytotoxicity), thus supporting the formulation of recommendations and occupational exposure limits by regulatory agencies.

## 5. Conclusions

The exposure of Portuguese firefighters to coarse, fine, and ultrafine PM, and its composition on PM-bound PAHs and metal(loid)s were, for the first time, evaluated in two prescribed fires. Firefighters’ exposure to total PM (1408.3 in Fire 1 and 342.5 µg/m^3^ in Fire 2) was well below the limit defined by OSHA (5.00 mg/m^3^; OSHA, 2017). Fine and ultrafine PM were the predominant fractions (1133.0 and 165.2 µg/m^3^ in Fire 1 versus 225.0 and 107.6 µg/m^3^ in Fire 2, respectively) and represented more than 90% of total PM. The concentrations of total PM-bound PAHs (3260.2 ng/m^3^ and 412.1 ng/m^3^ in Fires 1 and 2, respectively) were mostly distributed between the fine and ultrafine PM, with the low-molecular-weight compounds being the most predominant in the coarse (85.1–98.4% of total PAHs), fine (41.9–97.6%), and ultrafine (54.9–98.0%) PM. Firefighters’ exposure to total PM-bound PAHs, including (possible/probable) carcinogenic compounds (3260.2 ng/m^3^ in Fire 1 versus 412.1 ng/m^3^ in Fire 2) was well below the limits defined by OSHA (200 µg/m^3^; OSHA, 2017) and NIOSH (100 µg/m^3^; ATSDR, 2013). The levels of total PM-bound metals (660.8 ng/m^3^ in Fire 1 versus 262.2 ng/m^3^ in Fire 2) in the air of firefighters were also mostly distributed between the fine (46.9–63.6% of total PM) and ultrafine (18.0–34.2% of total PM) fractions of PM. The most abundant elements bound to PM were Zn, Cu, and Cr. During prescribed fires, firefighters were exposed to total (possible/probable) carcinogenic metals of 83.52 ng/m^3^ in Fire 1 and 39.79 ng/m^3^ in Fire 2 (11.7–12.6% of the total PM-bound metals). The TR levels for PM-bound PAHs were predominantly below the USEPA guidelines, while for PM-bound metals, the estimated values were 150–237 times higher than the recommended 10^−4^ level. This work characterizes firefighters’ exposure to PM, PM-bound PAHs, and metal(loid)s and the estimated health risks related to two controlled forest fire events that were performed in specific and non-replicable conditions, e.g., meteorologic conditions (temperature, relative humidity, precipitation, and wind speed/direction), terrain topology (i.e., inclination), and fuel available that is strongly conditioned by plants’ species, including its density and height. The results represent a live, true view of controlled forest-fire emissions, and more studies characterizing firefighting activities during controlled and principally real wildfires are needed to corroborate the findings presented in this study. Additionally, only three groups of pollutants, PM, PM-bound PAHs, and metal(loid)s, were monitored in this study. However, firefighters are exposed to other health-relevant pollutants released during fires, such as gas-phase semi-volatile and volatile organic compounds (alkanes and alkenes), phenols, and levoglucosan-derived species, among others, which also need to be explored in different firefighting scenarios, e.g., wildland fires [39,98]. Future studies assessing firefighters’ potential health risks (e.g., skin irritation and the development/aggravation of respiratory and cardiovascular diseases) due to exposure to PM released during fires and its content in PAHs and metals will be determinant to improve the characterization of occupational exposure and to propose mitigation procedures to reduce, as much as possible, the impact of fire effluents on firefighting forces.

## Figures and Tables

**Table 1:** Concentrations of PAHs (median and range; ng/m^3^) in coarse, fine, and ultrafine PM during prescribed fires.

Compound	Fire 1			Fire 2		
	Coarse	Fine	Ultrafine	Coarse	Fine	Ultrafine
Naphthalene	2.44 (2.28–2.55)	13.9 (13.1–14.7)	3.02 (2.88–3.16)	2.96 (2.81–3.05)	6.41 (6.06–6.74)	3.83 (3.60–4.07)
Acenaphthylene	83.7 (80.8–86.2)	832.9 (799.0–853.7)	219.7 (212.6–226.4)	93.7 (90.5–96.6)	191.9 (185.9–195.6)	99.4 (79.4–107.3)
Fluorene	0.979 (0.957–1.01)	17.5 (17.4–17.6)	3.30 (3.21–3.39)	0.795 (0.771–0.805)	2.40 (2.29–2.43)	1.20 (1.18–1.22)
Phenanthrene	1.79 (1.77–1.80)	236.4 (235.2–237.6)	12.2 (12.2–12.3)	0.192	0.50 (0.50–0.52)	0.256
Anthracene	0.0667	37.3 (36.6–38.2)	1.52 (1.39–1.53)	n.d.	n.d.	n.d.
Fluoranthene	7.69 (7.60–7.76)	523.0 (518.5–527.1)	58.9 (58.7–59.2)	0.164 (0.156–0.174)	0.668 (0.615–0.725)	0.181
Pyrene	6.20 (6.07–6.24)	379.0 (377.6–381.8)	40.5 (39.7–40.5)	0.176 (0.167–0.184)	0.617 (0.595–0.694)	0.160 (0.140–0.172)
Benz(a)anthracene	0.400 (0.389–0.421)	99.0 (97.6–101.3)	14.4 (14.3–14.6)	n.d.	n.d.	n.d.
Chrysene	0.230 (0.209–0.242)	88.3 (87.9–89.2)	13.1 (13.0–13.4)	0.341 (0.260–0.407)	0.919 (0.811–1.11)	0.194 (0.147–0.209)
Benzo(b + j)fluoranthene	0.350 (0.346–0.371)	135.0 (134.1–137.0)	21.4 (21.3–21.6)	n.d.	n.d.	n.d.
Benzo(k)fluoranthene	0.0564	27.7 (27.3–27.9)	4.08 (4.03–4.11)	n.d.	n.d.	n.d.
Benzo(a)pyrene	0.284 (0.278–0.293)	114.4 (114.0–115.2)	17.3 (17.3–17.4)	n.d.	n.d.	n.d.
Dibenzo(a,l)pyrene	n.d.	n.d.	n.d.	0.178	0.498 (0.491–0.520)	0.237
Dibenzo(a,h)anthracene	0.167	126.6 (126.2–126.9)	16.9 (16.6–17.1)	n.d.	n.d.	n.d.
Benzo(g,h,i)perylene	0.157	6.93 (6.79–7.00)	0.210	n.d.	n.d.	n.d.
Indeno(1,2,3-c,d)pyrene	0.0961	81.2 (79.5–82.6)	10.2 (9.72–10.5)	0.119	0.820 (0.780–0.923)	0.561 (0.537–0.568)
Total PAHs	104.6 (101.3–107.4)	2719.0 (2670.5–2757.7)	436.6 (427.1–445.3)	99.2 (95.7–102.3)	206.1 (199.4–210.6)	106.8 (86.4–115.0)
Total PAHs _carc_	4.18 (3.98–4.35)	692.9 (686.4–701.8)	100.6 (99.3–102.0)	3.99 (3.76–4.14)	9.48 (8.99–10.1)	5.35 (5.05–5.61)

n.d.—not detected in the fire event. When the concentrations determined were below the LOD, the value LOD/√2 was used [72].

**Table 2:** Concentrations of metals (median and range; ng/m^3^) in coarse, fine, and ultrafine PM during prescribed fires.

Element	Fire 1			Fire 2		
	Coarse	Fine	Ultrafine	Coarse	Fine	Ultrafine
Cr	23.5 (21.1–24.7)	62.8 (60.2–69.4)	27.4 (26.1–31.8)	11.5 (10.8–13.7)	32.5 (29.8–36.6)	23.2 (21.8–26.3)
Co	2.78 (2.67–2.83)	10.9 (10.6–11.1)	5.52 (5.38–5.61)	2.03 (2.00–2.07)	6.54 (6.45–6.64)	2.00 (1.93–2.04)
Ni	9.65 (9.58–9.94)	32.7 (31.5–34.3)	6.01 (5.44–6.29)	2.96 (2.78–3.09)	5.59 (5.40–5.69)	5.28 (4.94–5.48)
Cu	38.1 (37.2–38.64)	130.3 (129.0–132.8)	26.9 (26.1–27.3)	10.8 (10.5–11.8)	22.5 (22.3–22.7)	20.2 (19.9–20.6)
Zn	37.6 (37.2–38.8)	151.5 (149.3–153.8)	46.8 (46.0–47.5)	18.4 (18.3–18.4)	45.0 (44.7–45.5)	33.4 (30.2–33.7)
Sr	1.04 (0.892–1.60)	2.53 (2.36–3.27)	1.03 (0.791–1.18)	0.524 (0.520–0.662)	2.03 (1.92–2.41)	0.670 (0.492–0.921)
Mo	4.44 (4.35–4.48)	15.0 (14.7–15.3)	2.49 (2.44–2.54)	0.905 (0.673–1.50)	2.40 (2.40–2.41)	2.19 (2.15–2.30)
Cd	0.0198 (0.0193–0.0326)	0.146 (0.111–0.188)	0.0269 (0.0253–0.0330)	n.d.	0.0442 (0.0407–0.0514)	0.0189 (0.0157–0.0300)
Sb	0.203 (0.135–0.216)	0.398 (0.312–0.491)	0.165 (0.0965–0.228)	1.27 (1.26–1.28)	0.223 (0.185–0.285)	0.128 (0.105–0.177)
Cs	n.d.	0.204 (0.191–0.233)	0.0633 (0.0608–0.0653)	n.d.	0.0656 (0.0598–0.0664)	n.d.
Ba	1.14 (1.00–1.28)	2.08 (1.74–2.22)	0.854 (0.731–1.11)	0.0725 (0.0387–0.0889)	3.09 (2.99–3.41)	0.596 (0.440–0.692)
Tl	0.0547 (0.0510–0.0555)	0.172 (0.166–0.184)	n.d.	n.d.	0.0503 (0.0488–0.0537)	0.0300 (0.0297–0.0311)
Pb	2.80 (2.75–2.88)	10.5 (10.2–10.6)	1.76 (1.72–1.77)	0.234 (0.234–0.281)	2.46 (2.45–2.48)	1.50 (1.47–1.53)
Total metals	121.8 (117.3–125.9)	420.0 (411.2–434.8)	119.1 (115.0–125.5)	49.61 (48.00–53.93)	123.1 (119.2–128.8)	89.56 (83.89–94.22)
Total metals _carc_	15.45 (15.16–15.91)	54.58 (52.73–56.65)	13.49 (12.67–13.92)	7.017 (6.789–7.373)	14.85 (14.52–15.14)	8.924 (8.452–9.261)

n.d.—not detected in the fire event. When the concentrations determined were below the LOD, the value LOD/√2 was used [72].

## Data Availability

Data will be made available upon request.

## Supplementary Materials

The following supporting information can be downloaded at https://www.mdpi.com/article/10.3390/toxics12060422/s1, Figure S1: Graphical representation of the concentration of 14 PM fractions collected in Fire 1 and Fire 2; Table S1. Example of calculation of carcinogenic risk (TR) for naphthalene in Fire 1; Table S2: Concentrations of PM_2.5_ (median and range, µg/m^3^, except when indicated otherwise) during prescribed and wildland fires reported in the literature; Table S3: Concentrations of PAHs (median and range; ng/µg particles) in coarse, fine, and ultrafine PM during prescribed fires; Table S4: Concentrations of PM_2.5_-bound total PAHs (median and range, µg/m^3^, except when indicated otherwise) during prescribed and wildland fires reported in the literature; Table S5: Concentrations of metals (median and range; ng/µg particles) in coarse, fine, and ultrafine PM during prescribed fires; Table S6: Concentrations of PM_2.5_-bound metals (average ± standard deviation; µg/m^3^) during prescribed and wildland fires reported in the literature; Table S7: Levels of total TRs (median, range) estimated for firefighters enrolled in Fire 1 and Fire 2.
