# Supplementary material for: Characterization of Wildland Firefighters’ Exposure to Coarse, Fine, and Ultrafine Particles; Polycyclic Aromatic Hydrocarbons; and Metal(loid)s, and Estimation of Associated Health Risks
Source: Toxics. 2024 Jun 10;12(6):422. doi: 10.3390/toxics12060422 (PMC11209316; doi:10.3390/toxics12060422)
Supplement: Supplementary file 1 [file toxics-12-00422-s001.zip › toxics-3022415-supplementary.pdf]

Supplementary Material

Characterization of Wildland Firefighters' Exposure to Coarse, Fine, and Ultrafine Particles; Polycyclic Aromatic Hydrocarbons; and Metal(loid)s, and Estimation of Associated Health Risks

Joana Teixeira [1,2,†], Gabriel Sousa [1,†], Rui Azevedo [3], Agostinho Almeida [3], Cristina Delerue-Matos [1], Xianyu Wang [4], Alice Santos-Silva [2,5], Francisca Rodrigues [1] and Marta Oliveira [1,*]

[1] REQUIMTE/LAQV, Instituto Superior de Engenharia do Porto, Instituto Politécnico do Porto, R. Dr. António Bernardino de Almeida 431, 4249-015 Porto, Portugal; joana.teixeira@graq.isep.ipp.pt (J.T.); gabriel.sousa@graq.isep.ipp.pt (G.S.); cmm@isep.ipp.pt (C.D.-M.); francisca.rodrigues@graq.isep.ipp.pt (F.R.)

[2] REQUIMTE/UCIBIO, Unidade de Ciências Biomoleculares Aplicadas, Departamento de Ciências Biológicas, Faculdade de Farmácia, Universidade do Porto, R. Jorge de Viterbo Ferreira 228, 4050-313, Porto, Portugal; assilva@ff.up.pt

[3] REQUIMTE/LAQV, Departamento de Ciências Químicas, Faculdade de Farmácia, Universidade do Porto, R. Jorge Viterbo Ferreira, 228, 4050-313 Porto, Portugal; ruiazevedo43@gmail.com (R.A.); aalmeida@ff.up.pt (A.A.)

[4] QAEHS, Queensland Alliance for Environmental Health Sciences, The University of Queensland, 20 Cornwall Street, Woolloongabba, 4102, Queensland, Australia; x.wang18@uq.edu.au

[5] Laboratório Associado i4HB, Instituto para a Saúde e a Bioeconomia, Faculdade de Farmácia, Universidade do Porto, R. Jorge de Viterbo Ferreira 228, 4050-313, Porto, Portugal

* Correspondence: marta.oliveira@graq.isep.ipp.pt; Tel.: +351-22-834-0500

† Both authors contributed equally to the manuscript.

Table S1. Example of calculation of carcinogenic risk (TR) for naphthalene in Fire 1.

**Parameter**		**TR**
EF_R (days/year)	250	2.11×10^{-9}
ED (years)	15.4	
ET (h/day)	3.5 h *per* 24h, *i.e.*, 0.15	
AT (days)	14600 (40 years of career × 365 days/year)	
$C_{Naphthalene}$($\mu g/m^3$)	1.61×10^{-3}	
$IUR_{Naphthalene}$($\mu g/m^3$)$^{-1}$	3.40×10^{-5}	

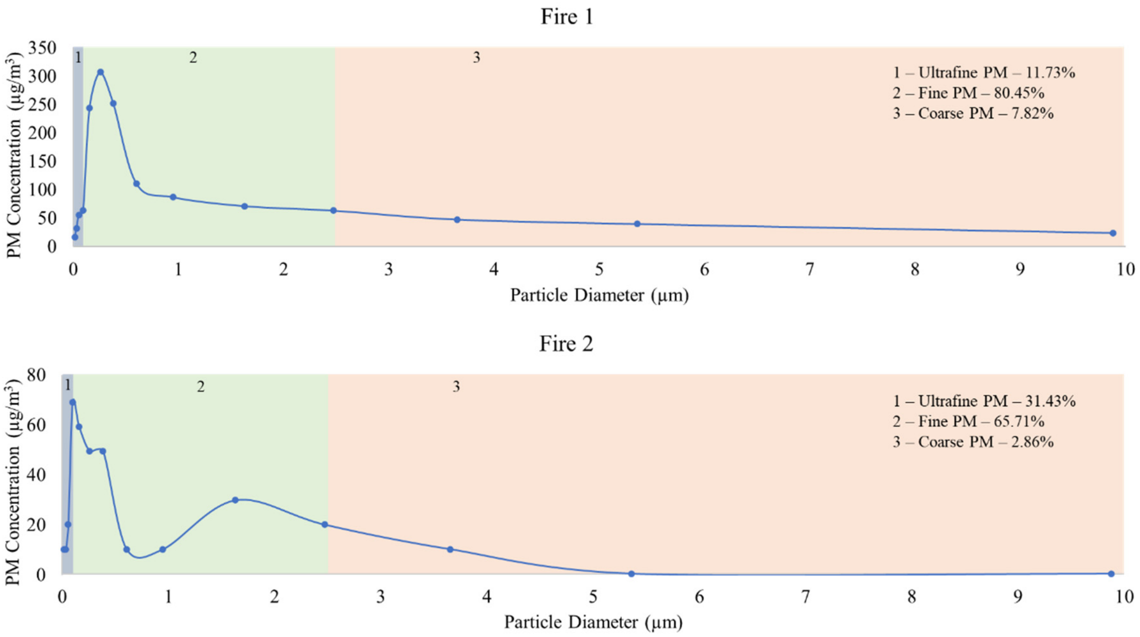

Figure S1. Graphical representation of the concentration of 14 PM fractions collected in Fire 1 and Fire 2.

Table S2. Concentrations of $PM_{2.5}$ (median and range, μg/m^3, except indicated otherwise) during prescribed and wildland fires reported in the literature.

Fire event (location)	Fuel composition	Sampling equipment	Concentrations	Reference
Prescribed fire (Savannah River Site, South Carolina, USA)	31% hardwood pine and 69% pine	Air pump combined with cyclone	264 (221 – 316)	[1]
			248 (184 – 333)	[2]
		Air pump with personal exposure monitor	259.4 (156.1 – 431.1)	[3]
Prescribed (Georgia, USA)	Mostly pine	Portable particulate sampler	1810 ± 680*	[4]
5 wildfires (Washington; Oregon; and California, USA)	N/S	Aerosol Monitor	8.8 - 41.3*	[5]
Wildfire (Sierra National Forest, California)	N/S	Environmental Beta Attenuation Monitor	12 – 49	[6]
Prescribed fires (southeastern USA)	N/S	Personal air sampling pump with cyclone	1054*	[7]
Prescribed fire (Savannah River Site, South Carolina, USA)	31% hardwood pine and 69% pine	Air pump combined with cyclone	650 (510 – 828)	[8]
			354 (313 – 400)	[9]
Prescribed fires (Australia)	N/S	Personal aerosol monitor	520 (20 – 16000)	[10]
21 prescribed fires (Apache-Sitgreaves National Forests, and Coconino National Forest Arizona)	Ponderosa pine, oak, Douglas fir, and mixed conifers	Speciation Sampler	523 – 8357*	[11]
Peat fire (Sumatra, Indonesia)	N/S	MiniVol portable air sampler	140 – 1600	[12]
8 prescribed fires (Wayne National Forest, Ohio, USA)	Oak (63%), maple (21%), and cottonwood (9%)	Air pump with personal exposure monitor	1750 ± 1200*	[13]

N/S – Not-specified

*Average (or Average ± Standard Deviation)

Table S3. Concentrations of PAHs (median and range; ng/μg particles) in coarse, fine, and ultrafine PM during prescribed fires.

Compound	Fire 1			Fire 2		
	Coarse	Fine	Ultrafine	Coarse	Fine	Ultrafine
Naphthalene	1.48×10^{-2} (1.38×10^{-2} – 1.54×10^{-2})	1.23×10^{-2} (1.16×10^{-2} – 1.30×10^{-2})	2.74×10^{-2} (2.62×10^{-2} – 2.87×10^{-2})	2.75×10^{-2} (2.61×10^{-2} – 2.83×10^{-2})	2.85×10^{-2} (2.69×10^{-2} – 2.99×10^{-2})	0.391 (0.368 – 0.416)
Acenaphthylene	0.506 (0.489 – 0.522)	0.735 (0.705 – 0.753)	1.99 (1.93 – 2.06)	0.871 (0.840 – 0.897)	0.853 (0.826 – 0.869)	10.2 (8.11 – 11.0)
Fluorene	5.92×10^{-3} (5.79×10^{-3} – 6.12×10^{-3})	1.54×10^{-2} (1.53×10^{-2} – 1.56×10^{-2})	2.99×10^{-2} (2.91×10^{-2} – 3.08×10^{-2})	7.39×10^{-3} (7.16×10^{-3} – 7.48×10^{-3})	1.07×10^{-2} (1.02×10^{-2} – 1.08×10^{-2})	0.122 (0.121 – 0.124)
Phenanthrene	1.09×10^{-2} (1.07×10^{-2} – 1.09×10^{-2})	0.209 (0.208 – 0.210)	0.111 (0.110 – 0.112)	1.79×10^{-3}	2.22×10^{-3} (2.21×10^{-3} – 2.30×10^{-3})	2.62×10^{-2}
Anthracene	4.04×10^{-4}	3.29×10^{-2} (3.23×10^{-2} – 3.37×10^{-2})	1.38×10^{-2} (1.27×10^{-2} – 1.39×10^{-2})	n.d.	n.d.	n.d.
Fluoranthene	4.66×10^{-2} (4.60×10^{-2} – 4.70×10^{-2})	0.462 (0.458 – 0.465)	0.535 (0.533 – 0.537)	1.53×10^{-3} (1.45×10^{-3} – 1.62×10^{-3})	2.97×10^{-3} (2.73×10^{-3} – 3.22×10^{-3})	1.85×10^{-2}
Pyrene	3.75×10^{-2} (3.67×10^{-2} – 3.78×10^{-2})	0.335 (0.333 – 0.337)	0.367 (0.360 – 0.368)	1.63×10^{-3} (1.55×10^{-3} – 1.71×10^{-3})	2.74×10^{-3} (2.64×10^{-3} – 3.08×10^{-3})	1.63×10^{-2} (1.44×10^{-2} – 1.76×10^{-2})
Benz(a)anthracene	2.42×10^{-3} (2.35×10^{-3} – 2.55×10^{-3})	8.74×10^{-2} (8.62×10^{-2} – 8.94×10^{-2})	0.131 (0.130 – 0.132)	n.d.	n.d.	n.d.
Chrysene	1.39×10^{-3} (1.27×10^{-3} – 1.46×10^{-3})	7.79×10^{-2} (7.76×10^{-2} – 7.87×10^{-2})	0.119 (0.118 – 0.122)	3.16×10^{-3} (2.41×10^{-3} – 3.78×10^{-3})	4.08×10^{-3} (3.60×10^{-3} – 4.92×10^{-3})	1.98×10^{-2} (1.50×10^{-2} – 2.13×10^{-2})
Benzo(b+j)fluoranthene	2.12×10^{-3} (2.09×10^{-3} – 2.24×10^{-3})	0.119 (0.0118 – 0.0121)	0.194 (0.193 – 0.196)	n.d.	n.d.	n.d.
Benzo(k)fluoranthene	3.42×10^{-4}	2.44×10^{-2} (2.41×10^{-2} – 2.46×10^{-2})	3.70×10^{-2} (3.66×10^{-2} – 3.74×10^{-2})	n.d.	n.d.	n.d.
Benzo(a)pyrene	1.72×10^{-3} (1.69×10^{-3} – 1.77×10^{-3})	0.101 (0.101 – 0.102)	0.157 (0.157 – 0.158)	n.d.	n.d.	n.d.
Dibenzo(a,l)pyrene	n.d.	n.d.	n.d.	1.65×10^{-3}	2.21×10^{-3} (2.18×10^{-3} – 2.31×10^{-3})	2.42×10^{-2}
Dibenzo(a,h)anthracene	1.01×10^{-3}	0.112 (0.111 – 0.112)	0.154 (0.151 – 0.155)	n.d.	n.d.	n.d.
Benzo(g,h,i)perylene	9.53×10^{-4}	6.11×10^{-3} (5.99×10^{-3} – 6.17×10^{-3})	1.91×10^{-3}	n.d.	n.d.	n.d.
Indeno(1,2,3-c,d)pyrene	5.81×10^{-4}	7.16×10^{-2} (7.01×10^{-2} – 7.29×10^{-2})	9.23×10^{-2} (8.82×10^{-2} – 9.50×10^{-2})	1.11×10^{-3}	3.64×10^{-3} (3.46×10^{-3} – 4.10×10^{-3})	5.74×10^{-2} (5.49×10^{-2} – 5.81×10^{-2})

Total PAHs	0.633 (0.613 – 0.650)	2.40 (2.36 – 2.43)	3.96 (3.88 – 4.04)	0.922 (0.889 – 0.950)	0.916 (0.886 – 0.936)	10.9 (8.83 – 11.7)
Total PAHs $_{carc}$	2.53×10^{-2} (2.41×10^{-2} – 2.63×10^{-2})	0.612 (0.606 – 0.619)	0.913 (0.902 – 0.926)	3.71×10^{-2} (3.49×10^{-2} – 3.85×10^{-2})	4.21×10^{-2} (3.40×10^{-2} – 4.49×10^{-2})	0.546 (0.516 – 0.573)

n.d. – Not-detected

Table S4. Concentrations of $PM_{2.5}$-bound total PAHs (median and range, μg/m^3, except indicated otherwise) during prescribed and wildland fires reported in the literature.

Fire event (location)	Fuel composition	Concentration	Reference
Prescribed shrubland fire (Ayora, Valencia, Spain)	30-year-old shrubland vegetation (Mediterranean gorse and rosemary)	≈1.40	[14]
Prescribed and wildfire (Klamath River, and Sierra National Forest, California)	N/S	0.265 – 0.586	[15]
Wildfire (Sierra National Forest, California)	N/S	0.040 ± 0.002 to 0.045 ± 0.002*	[6]
5 prescribed pile burns (Apache–Sitgreaves National Forest, Arizona)	Ponderosa pine slash, oak, and Douglas fir	7.64 (4.61 – 10.78)	[16]
21 prescribed fires (Apache-Sitgreaves National Forests, and Coconino National Forest Arizona)	Ponderosa pine, oak, Douglas fir, and mixed conifers	0.69 ± 0.37 to 2.24 ± 0.23[a*]	[11]
Peat fire (Sumatra, Indonesia)	N/S	0.135 – 0.562	[12]
Wildfire (Alberta, Canada)	N/S	0.852 (0.300 – 0.980)	[17]

N/S – Not-specified

[a] – Values presented in mg PAH/gOC (Organic carbon).

*average ± standard deviation

Table S5. Concentrations of metals (median and range; ng/µg particles) in coarse, fine, and ultrafine PM during prescribed fires.

Element	Fire 1			Fire 2		
	Coarse	Fine	Ultrafine	Coarse	Fine	Ultrafine
Cr	0.142<br>(0.127 – 0.149)	5.54×10^{-2}<br>(5.32×10^{-2} – 6.13×10^{-2})	0.249<br>(0.237 – 0.289)	0.214<br>(0.201 – 0.255)	0.289<br>(0.265 – 0.325)	4.75<br>(4.47 – 5.38)
Co	1.56×10^{-2}<br>(1.49×10^{-2} – 1.59×10^{-2})	0.00941×10^{-3}<br>(0.00918 – 0.00957)	5.01×10^{-2}<br>(4.88×10^{-2} – 5.09×10^{-2})	3.53×10^{-2}<br>(3.48×10^{-2} – 3.60×10^{-2})	5.47×10^{-2}<br>(5.39×10^{-2} – 5.55×10^{-2})	0.408<br>(0.394 – 0.417)
Ni	5.46×10^{-2}<br>(5.42×10^{-2} – 5.64×10^{-2})	2.78×10^{-2}<br>(2.67×10^{-2} – 2.92×10^{-2})	5.46×10^{-2}<br>(4.94×10^{-2} – 5.71×10^{-2})	4.78×10^{-2}<br>(4.44×10^{-2} – 5.02×10^{-2})	3.24×10^{-2}<br>(3.08×10^{-2} – 3.33×10^{-2})	1.08<br>(1.01 – 1.12)
Cu	0.216<br>(0.211 – 0.220)	0.111<br>(0.110 – 0.113)	0.244<br>(0.237 – 0.248)	0.148<br>(0.142 – 0.153)	0.136<br>(0.134 – 0.138)	4.13<br>(4.06 – 4.22)
Zn	0.177<br>(0.174 – 0.184)	0.126<br>(0.124 – 0.128)	0.425<br>(0.418 – 0.431)	0.148<br>(0.146 – 0.148)	0.122<br>(0.119 – 0.127)	6.83<br>(6.18 – 6.89)
Sr	5.08×10^{-3}<br>(4.20×10^{-3} – 7.69×10^{-3})	1.89×10^{-3}<br>(1.73×10^{-3} – 2.35×10^{-3})	5.79×10^{-3}<br>(3.59×10^{-3} – 6.19×10^{-3})	5.16×10^{-3}<br>(5.09×10^{-3} – 7.74×10^{-3})	1.26×10^{-2}<br>(1.16×10^{-2} – 1.32×10^{-2})	8.68×10^{-2}<br>(5.03×10^{-2} – 0.103)
Mo	2.51×10^{-2}<br>(2.45×10^{-2} – 2.53×10^{-2})	1.27×10^{-2}<br>(1.24×10^{-2} – 1.30×10^{-2})	1.72×10^{-2}<br>(1.68×10^{-2} – 1.77×10^{-2})	5.60×10^{-3}<br>(5.60×10^{-3} – 1.67×10^{-2})	1.31×10^{-2}<br>(1.31×10^{-2} – 1.32×10^{-2})	0.409<br>(0.402 – 0.415)
Cd	5.12×10^{-5}<br>(4.87×10^{-5} – 7.61×10^{-5})	1.24×10^{-4}<br>(8.77×10^{-5} – 1.45×10^{-4})	9.07×10^{-5}<br>(7.61×10^{-5} – 1.45×10^{-4})	n.d.	2.06×10^{-4}<br>(1.74×10^{-4} – 2.69×10^{-4})	2.43×10^{-2}<br>(1.77×10^{-3} – 3.60×10^{-3})
Sb	1.23×10^{-3}<br>(8.17×10^{-4} – 1.31×10^{-3})	3.33×10^{-4}<br>(2.57×10^{-4} – 4.15×10^{-4})	1.31×10^{-3}<br>(6.88×10^{-4} – 1.50×10^{-3})	2.02×10^{-2}<br>(1.99×10^{-2} – 2.02×10^{-2})	1.75×10^{-3}<br>(1.41×10^{-3} – 2.13×10^{-3})	2.61×10^{-2}<br>(2.14×10^{-2} – 3.62×10^{-2})
Cs	n.d.	1.36×10^{-4}<br>(1.24×10^{-4} – 1.54×10^{-4})	2.34×10^{-4}<br>(2.11×10^{-4} – 2.51×10^{-4})	n.d.	1.68×10^{-4}<br>(1.16×10^{-4} – 1.75×10^{-4})	n.d.
Ba	6.90×10^{-3}<br>(6.07×10^{-3} – 7.74×10^{-3})	1.56×10^{-3}<br>(1.27×10^{-3} – 1.69×10^{-3})	4.94×10^{-3}<br>(3.82×10^{-3} – 6.59×10^{-3})	6.14×10^{-4}<br>(2.40×10^{-4} – 8.95×10^{-4})	2.40×10^{-2}<br>(2.31×10^{-2} – 2.58×10^{-2})	4.64×10^{-2}<br>(4.64×10^{-2} – 0.137)
Tl	2.69×10^{-4}<br>(2.47×10^{-4} – 2.73×10^{-4})	1.25×10^{-4}<br>1.19×10^{-4} – 1.30×10^{-4})	n.d.	n.d.	10.5×10^{-5}<br>(9.23×10^{-5} – 13.6×10^{-5})	2.21×10^{-3}<br>(2.13×10^{-3} – 2.43×10^{-3})
Pb	1.57×10^{-2}<br>(1.54×10^{-2} – 1.62×10^{-2})	9.06×10^{-3}<br>(8.86×10^{-3} – 9.16×10^{-3})	1.23×10^{-2}<br>(1.20×10^{-2} – 1.24×10^{-2})	1.45×10^{-3}<br>(1.45×10^{-3} – 2.32×10^{-3})	1.63×10^{-2}<br>(1.62×10^{-2} – 1.65×10^{-2})	0.307<br>(0.307 – 0.475)
Total metals	0.660	0.356	1.06	0.626	0.702	18.1

	(0.633 – 0.684)	(0.348 – 0.368)	(1.03 – 1.12)	(0.600 – 0.690)	(0.668 – 0.750)	(16.9 – 19.2)
Total metals $_{carc}$	8.72×10^{-2} (8.54×10^{-2} – 8.99×10^{-2})	4.67×10^{-2} (4.51×10^{-2} – 4.85×10^{-2})	0.118 (0.111 – 0.122)	0.105 (0.101 – 0.109)	0.105 (0.102 – 0.108)	1.82 (1.73 – 2.05)

n.d. – not-detected

Table S6. Concentrations of $PM_{2.5}$-bound metals (average ± standard deviation; μg/m^3) during prescribed and wildland fires reported in the literature.

Event and Location	Fuel composition	Concentration	Reference
Prescribed fire (Georgia, USA)	Mostly pine	22.29 ± 5.221	[4]
8 prescribed fires (Arizona, USA)	Mostly ponderosa pine	7.972 ± 2.07<br>30.41 ± 1.094	[18]
21 prescribed fires (Apache-Sitgreaves National Forests, and Coconino National Forest Arizona)	Ponderosa pine, oak, Douglas fir, and mixed conifers	2.311 - 11.60	[11]
Peat fire (Sumatra, Indonesia)	N/S	54.10 ± 26.42<br>14.52 ± 6.207	[12]
8 prescribed fires (Wayne National Forest, Ohio, USA)	Oak (63%), maple (21%), and cottonwood (9%)	18.71 ± 1.454	[13]

N/S – Not-specified

Table S7. Levels of total TRs (median, range) estimated for firefighters enrolled in Fire 1 and Fire 2.

	**Fire 1**			**Fire 2**		
	Coarse	Fine	Ultrafine	Coarse	Fine	Ultrafine
**PM-bound PAHs**	1.08×10^{-8} (9.52×10^{-9} – 1.09×10^{-8})	1.47×10^{-6} (1.71×10^{-8} – 4.39×10^{-6})	3.04×10^{-7} (3.54×10^{-8} – 1.24×10^{-6})	7.68×10^{-9} (6.97×10^{-9} – 8.10×10^{-9})	8.10×10^{-9} (6.97×10^{-9} – 8.97×10^{-9})	8.16×10^{-9} (7.98×10^{-9} – 8.73×10^{-9})
**PM-bound metal(loid)s**	2.71×10^{-2} (1.63×10^{-2} – 3.37×10^{-2})	2.58×10^{-2} (1.14×10^{-2} – 5.05×10^{-2})	2.33×10^{-2} (1.96×10^{-2} – 2.42×10^{-2})	1.09×10^{-2} (9.64×10^{-3} – 1.74×10^{-2})	1.41×10^{-2} (1.17×10^{-2} – 2.43×10^{-2})	1.89×10^{-2} (1.73×10^{-2} – 2.06×10^{-2})

References

1. Adetona, O.; Hall, D.B.; Naeher, L.P. Lung function changes in wildland firefighters working at prescribed burns. *Inhal. Toxicol.* **2011**, *23*, 835-841, doi:10.3109/08958378.2011.617790.
2. Adetona, O.; Zhang, J.J.; Hall, D.B.; Wang, J.S.; Vena, J.E.; Naeher, L.P. Occupational exposure to woodsmoke and oxidative stress in wildland firefighters. *Sci. Total Environ.* **2013**, *449*, 269-275, doi:10.1016/j.scitotenv.2013.01.075.
3. Adetona, A.M.; Kyle Martin, W.; Warren, S.H.; Hanley, N.M.; Adetona, O.; Zhang, J.; Simpson, C.; Paulsen, M.; Rathbun, S.; Wang, J.-S.; et al. Urinary mutagenicity and other biomarkers of occupational smoke exposure of wildland firefighters and oxidative stress. *Inhal. Toxicol.* **2019**, *31*, 73-87, doi:10.1080/08958378.2019.1600079.
4. Lee, S.; Baumann, K.; Schauer, J.J.; Sheesley, R.J.; Naeher, L.P.; Meinardi, S.; Blake, D.R.; Edgerton, E.S.; Russell, A.G.; Clements, M. Gaseous and Particulate Emissions from Prescribed Burning in Georgia. *Environ. Sci. Technol.* **2005**, *39*, 9049-9056, doi:10.1021/es051583l.
5. McNamara, M.L.; Semmens, E.O.; Gaskill, S.; Palmer, C.; Noonan, C.W.; Ward, T.J. Base Camp Personnel Exposure to Particulate Matter During Wildland Fire Suppression Activities. *J. Occup. Environ. Hyg.* **2012**, *9*, 149-156, doi:10.1080/15459624.2011.652934.
6. Navarro, K.M.; Cisneros, R.; Schweizer, D.; Chowdhary, P.; Noth, E.M.; Balmes, J.R.; Hammond, S.K. Incident command post exposure to polycyclic aromatic hydrocarbons and particulate matter during a wildfire. *J. Occup. Environ. Hyg.* **2019**, *16*, 735-744, doi:10.1080/15459624.2019.1657579.
7. Neitzel, R.; Naeher, L.P.; Paulsen, M.; Dunn, K.; Stock, A.; Simpson, C.D. Biological monitoring of smoke exposure among wildland firefighters: A pilot study comparing urinary methoxyphenols with personal exposures to carbon monoxide, particular matter, and levoglucosan. *J. Expo. Sci. Env. Epid.* **2009**, *19*, 349-358, doi:10.1038/jes.2008.21.
8. Hejl, A.M.; Adetona, O.; Diaz-Sanchez, D.; Carter, J.D.; Commodore, A.A.; Rathbun, S.L.; Naeher, L.P. Inflammatory Effects of Woodsmoke Exposure Among Wildland Firefighters Working at Prescribed Burns at the Savannah River Site, SC. *J. Occup. Environ. Hyg.* **2013**, *10*, 173-180, doi:10.1080/15459624.2012.760064.
9. Naeher, L.P.; Barr, D.B.; Adetona, O.; Simpson, C.D. Urinary levoglucosan as a biomarker for woodsmoke exposure in wildland firefighters. *Int. J. Occup. Env. Heal.* **2013**, *19*, 304-310, doi:10.1179/2049396713Y.0000000037.
10. Reisen, F.; Hansen, D.; Meyer, C.P. Exposure to bushfire smoke during prescribed burns and wildfires: Firefighters' exposure risks and options. *Environ. Int.* **2011**, *37*, 314-321, doi:10.1016/j.envint.2010.09.005.
11. Robinson, M.S.; Zhao, M.; Zack, L.; Brindley, C.; Portz, L.; Quarterman, M.; Long, X.; Herckes, P. Characterization of PM2.5 collected during broadcast and slash-

pile prescribed burns of predominately ponderosa pine forests in northern Arizona. *Atmos. Environ.* **2011**, *45*, 2087-2094, doi:10.1016/j.atmosenv.2011.01.051.
12. See, S.W.; Balasubramanian, R.; Rianawati, E.; Karthikeyan, S.; Streets, D.G. Characterization and Source Apportionment of Particulate Matter ≤ 2.5 μm in Sumatra, Indonesia, during a Recent Peat Fire Episode. *Environ. Sci. Technol.* **2007**, *41*, 3488-3494, doi:10.1021/es061943k.
13. Wu, C.-M.; Song, C.; Chartier, R.; Kremer, J.; Naeher, L.; Adetona, O. Characterization of occupational smoke exposure among wildland firefighters in the midwestern United States. *Environ. Res.* **2021**, *193*, 110541-110550, doi:10.1016/j.envres.2020.110541.
14. Garcia-Hurtado, E.; Pey, J.; Borrás, E.; Sánchez, P.; Vera, T.; Carratalá, A.; Alastuey, A.; Querol, X.; Vallejo, V.R. Atmospheric PM and volatile organic compounds released from Mediterranean shrubland wildfires. *Atmos. Environ.* **2014**, *89*, 85-92, doi:10.1016/j.atmosenv.2014.02.016.
15. Navarro, K.M.; Cisneros, R.; Noth, E.M.; Balmes, J.R.; Hammond, S.K. Occupational Exposure to Polycyclic Aromatic Hydrocarbon of Wildland Firefighters at Prescribed and Wildland Fires. *Environ. Sci. Technol.* **2017**, *51*, 6461-6469, doi:10.1021/acs.est.7b00950.
16. Robinson, M.S.; Anthony, T.R.; Littau, S.R.; Herckes, P.; Nelson, X.; Poplin, G.S.; Burgess, J.L. Occupational PAH exposures during prescribed pile burns. *Ann. Occup. Hyg.* **2008**, *52*, 497-508, doi:10.1093/annhyg/men027.
17. Wentworth, G.R.; Aklilu, Y.-A.; Landis, M.S.; Hsu, Y.-M. Impacts of a large boreal wildfire on ground level atmospheric concentrations of PAHs, VOCs and ozone. *Atmos. Environ.* **2018**, *178*, 19-30, doi:10.1016/j.atmosenv.2018.01.013.
18. Robinson, M.S.; Chavez, J.; Velazquez, S.; Jayanty, R.K.M. Chemical Speciation of PM2.5 Collected during Prescribed Fires of the Coconino National Forest near Flagstaff, Arizona. *J. Air Waste Manage.* **2004**, *54*, 1112-1123, doi:10.1080/10473289.2004.10470985.
